# I-125 seeds brachytherapy combined with immunotherapy for MET amplification in non-small cell lung cancer from clinical application to related lncRNA mechanism explore: a case report

**DOI:** 10.3389/fcell.2023.1176083

**Published:** 2023-06-14

**Authors:** Mingxing Yang, Yuanli You, Xiuqing Wang, Wen Dong

**Affiliations:** Department of Respiratory Medicine, Hainan Cancer Hospital, Haikou, Hainan, China

**Keywords:** immunotherapy, MET aberration, non-small cell lung cancer, brachytherapy, outcome

## Abstract

Advanced non-small cell lung cancer (NSCLC) with MET amplification primarily relies on MET inhibitors for treatment, but once resistance occurs, the available treatment options are limited and the prognosis is typically poor. A 57-year-old man with advanced NSCLC and C-MET amplification was initially treated with crizotinib but developed progressive disease. After the antirotinib treatment, he achieved a partial response for a year. Genetic testing showed high PD-L1 expression, and he was treated with pembrolizumab and chemotherapy for 3 months, with partial response. Maintenance therapy with pembrolizumab and local I-125 seeds brachytherapy (ISB) was given after the lung lesion progressed but other lesions remained stable. The therapy resulted in significant resolution of the right upper lung lesion. It demonstrates the effectiveness of ISB-ICI combination in treating MET amplification advanced NSCLC. Ongoing research and treatment innovation are important in managing advanced NSCLC with complex genetic aberrations. To explore the candidate mechanism of ISB therapy response, we download public genetic data and conduct different expression Lncrnas analysis and pathway analysis to discover radiotherapy related sensitive or resistance lncRNAs and pathways, we found that AL654754.1 is a key lncRNA with radiotherapy response, and it also include in classical p53 and Wnt signaling pathway. Overall, the clinical case reports, combined with the exploration of underlying mechanisms, provide positive guidance for the precise treatment of lung cancer.

## Introduction

Globally, lung cancer is the most prevalent and deadly cancer, with 2.2 million new cases and 1.8 million deaths in 2020 (Sung et al., 2021). Non-small cell lung cancer (NSCLC) accounts for 85% of all lung cancers (Arriagada et al., 2010). While mesenchymal-epithelial transition factor (MET) amplification is a rare mutation, occurring in only 1%–5% of stage IV driver-positive NSCLC patients (Fujino et al., 2020), it is significant in terms of targeted therapy (Fujino et al., 2020). MET-targeting drugs, including kinase inhibitors and monoclonal antibodies, have been developed for this purpose. Among these, crizotinib was the first MET-targeted therapy recommended by the National Comprehensive Cancer Network (NCCN) clinical practice guidelines. However, crizotinib only achieved a 32% objective response rate (ORR) and 7.3 months of progression-free survival (PFS) (Drilon et al., 2020). In contrast, capmatinib has better anti-tumor activity than other MET inhibitors (Fujino et al., 2018) and was the first MET inhibitor to be approved by the US Food and Drug Administration (FDA) in May 2020. Nevertheless, resistance to single-agent tyrosine kinase inhibitors (TKI)-targeted therapy is inevitable (Reungwetwattana et al., 2017). When patients develop resistance to MET inhibitors, the available treatment options are limited and the prognosis is poor. Recently, inhibitors have made breakthrough progress in the treatment of NSCLC, but the therapeutic effect for patients with MET amplification is unclear (Joshua et al., 2018). In addition, there are few reports on local treatment strategies for advanced NSCLC patients who have developed resistance to MET inhibitors. I-125 seeds brachytherapy is a type of radiation therapy often used for cancer treatment. This method involves the direct implantation of radioactive substances into tumor tissues to treat cancer. Since the treatment is applied directly to the tumor, it can precisely deliver radiation doses to the tumor, minimizing impact on surrounding healthy tissues. In addition, as brachytherapy reduces radiation to healthy tissues, it also reduces the risk of some side effects associated with radiation therapy. This approach has been proven effective in the treatment of various types of cancer, including prostate cancer, liver cancer, and brain tumors. However, there are currently no reported cases of its use in the treatment of patients with MET mutation resistance (Chen et al., 2022).

**FIGURE 1:**
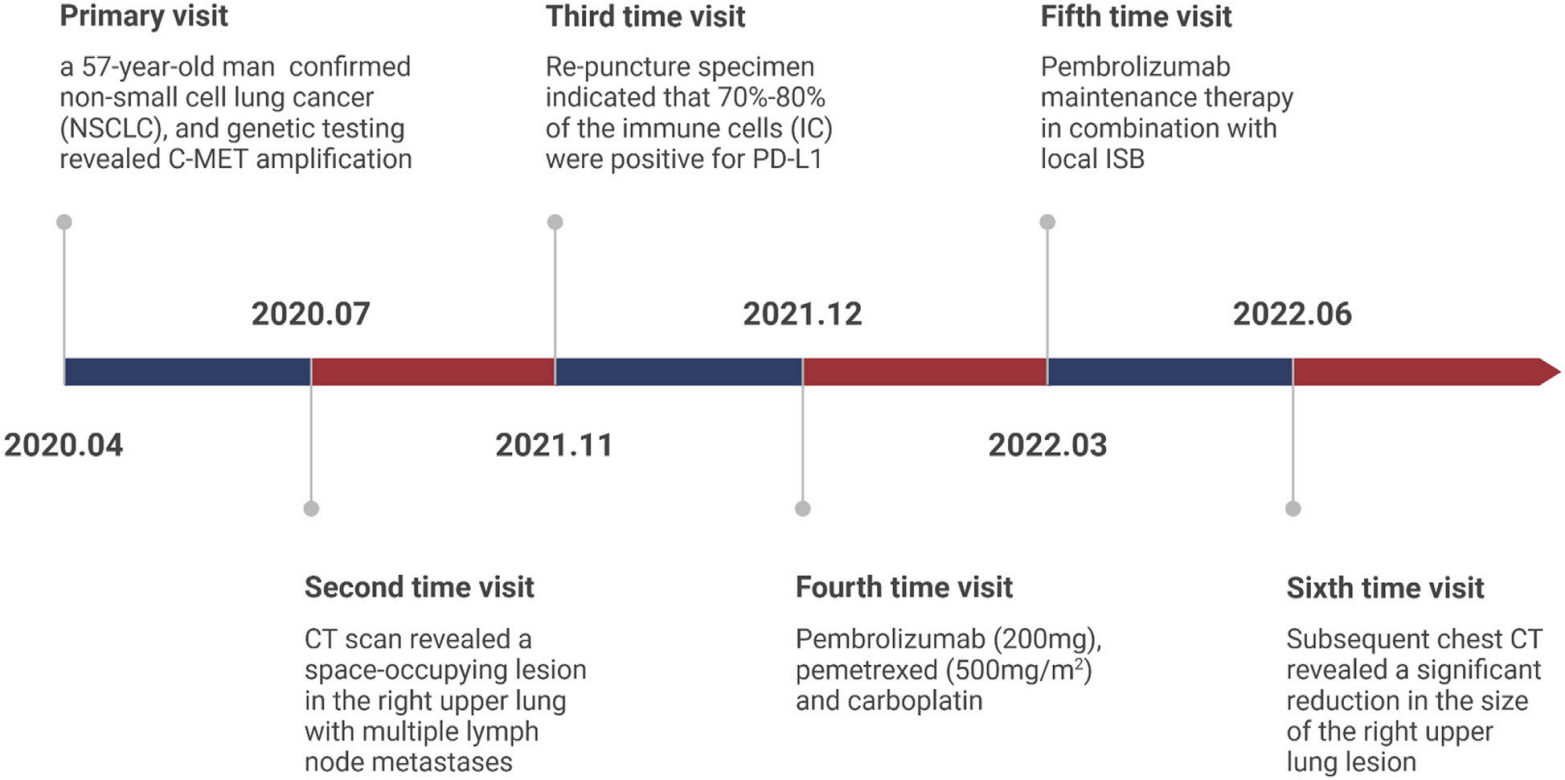
The detail information of Patient’s medical timeline.

Herein, we report a case of a patient diagnosed as NSCLC with MET amplification who responded well to a combination of ISB and immune checkpoint inhibitors (ICIs) and the medical timeline as list in Figure 1.

## Case report

In April 2020, a 57-year-old man presented at the Second Affiliated Hospital of Hainan Medical University with a persistent cough for 2 months. The patient had hepatitis C 2 years ago and had a smoking history of more than 40 years, a smoking index >800, and abstained for 1 year. At present, for hepatitis C, patients accept sofosbuvir with velpatasvir therapy with 3 months and turned negativeChest and abdomen computed tomography (CT) revealed a space-occupying lesion in the right upper lung, with multiple lymph node metastases and right adrenal gland metastases. Pathological biopsy confirmed non-small cell lung cancer (NSCLC), and genetic testing revealed C-MET amplification. The disease was staged as cT3N1M1b stage IVA, according to the eighth edition of the American Joint Committee on Cancer (AJCC). The patient’s general status score was 2 and with no family history of lung cancer.

The patient was treated with crizotinib (250 mg) targeted therapy, but the efficacy was assessed as progressive disease (PD) on chest CT review in July 2020 (Antitumor effectiveness was assessed by the independent review committee according to Response Evaluation Criteria in Solid Tumors v1.1.15). Consequently, antirotinib was administrated, the efficacy was evaluated as partial response (PR) and the treatment was continuous.

On 23 November 2021, the patient was readmitted to our hospital due to cough and chest pain. CT scan revealed a space-occupying lesion in the right upper lung with multiple lymph node metastases in the mediastinum, right supraclavicular, and hilar regions. Multiple small enlarged lymph nodes were also detected in the liver hilum, retroperitoneum, and mesentery. The patient underwent a re-biopsy to facilitate better treatment decisions. The lung alveolar tissue sent for examination showed epithelial hyperplasia, with some cells exhibiting vacuolation and foaming, widened alveolar septa, significant local fibrosis, and a large number of inflammatory cells, mainly lymphocytes, infiltrating the tissue. Taken together with the patient’s medical history, these findings are consistent with post-treatment changes in the tumor. The genetic testing results of the re-puncture specimen indicated that 70%–80% of the immune cells (IC) were positive for PD-L1.

The high expression of PD-L1 suggests that anti-PD-1 immunotherapy may be a suitable treatment option. Given the patient’s history of hepatitis C, Epclusa was administered for 1 month to prevent hepatitis C exacerbation, and a non-RNA copy number was detected after anti-hepatitis C treatment. On 24 December 2021, Pembrolizumab (200 mg), pemetrexed (500 mg/m^2^), and carboplatin (area under curve = 6) treatment was initiated and evaluated as a partial response (PR). However, after 4 cycles, the CT reexamination showed a larger lesion in the lung while the other lesion remained stable. As a result, Pembrolizumab maintenance therapy in combination with local ISB was initiated on 24 March 2022. No significant side effects were observed during treatment.

**FIGURE 2:**
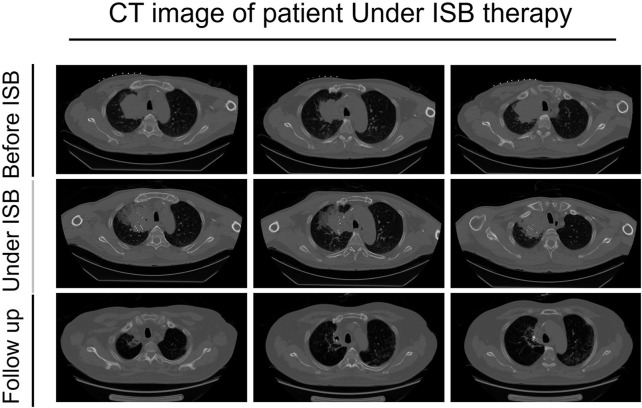
CT images of the patient before using ISB treatment, under the treatment, and follow-up after the treatment.

On 30 June 2022, the patient presented to our hospital with a dry cough. A subsequent chest CT revealed a significant reduction in the size of the right upper lung lesion, but a large solid shadow was observed in the right lung (Figure 2). Given the patient’s medical history, radiation pneumonia or immune pneumonia was considered, and therefore, immunotherapy was suspended on 8 July 2022. The patient was treated with ceftazidime injection combined with methylprednisolone (40 mg, once daily, 4 mg/tablet) for 3 days, resulting in less severe pneumonia on follow-up CT. Methylprednisolone was reduced to 32 mg once per week. The patient was administrated to end star and maintained on pemetrexed.

On 12 August 2022, methylprednisolone was further reduced to 16 mg once daily, but the patient had a fever (>39°C). A follow-up chest CT on 19 August revealed large consolidation shadows in both lungs, prompting an increase in methylprednisolone to 120 mg once daily. A subsequent CT scan 3 days later showed no significant changes. On 23 August, methylprednisolone was increased to 160 mg once daily, and the patient was given 10 g of human immunoglobulin over 3 days, in addition to mycophenolate mofetil capsules (0.5 g, twice daily). Infliximab (200 mg ivgtt) was added on 24 August.

On 29 August, CT indicated an improvement in pulmonary exudation, and methylprednisolone was reduced to 120 mg/day. A re-examination on 6 September showed no significant change, and the patient received a second dose of infliximab on 7 September, with methylprednisolone reduced to 80 mg/day. Methylprednisolone was then reduced to 40 mg/day on 10 September, followed by 4 mg every 2 weeks.

**FIGURE 3:**
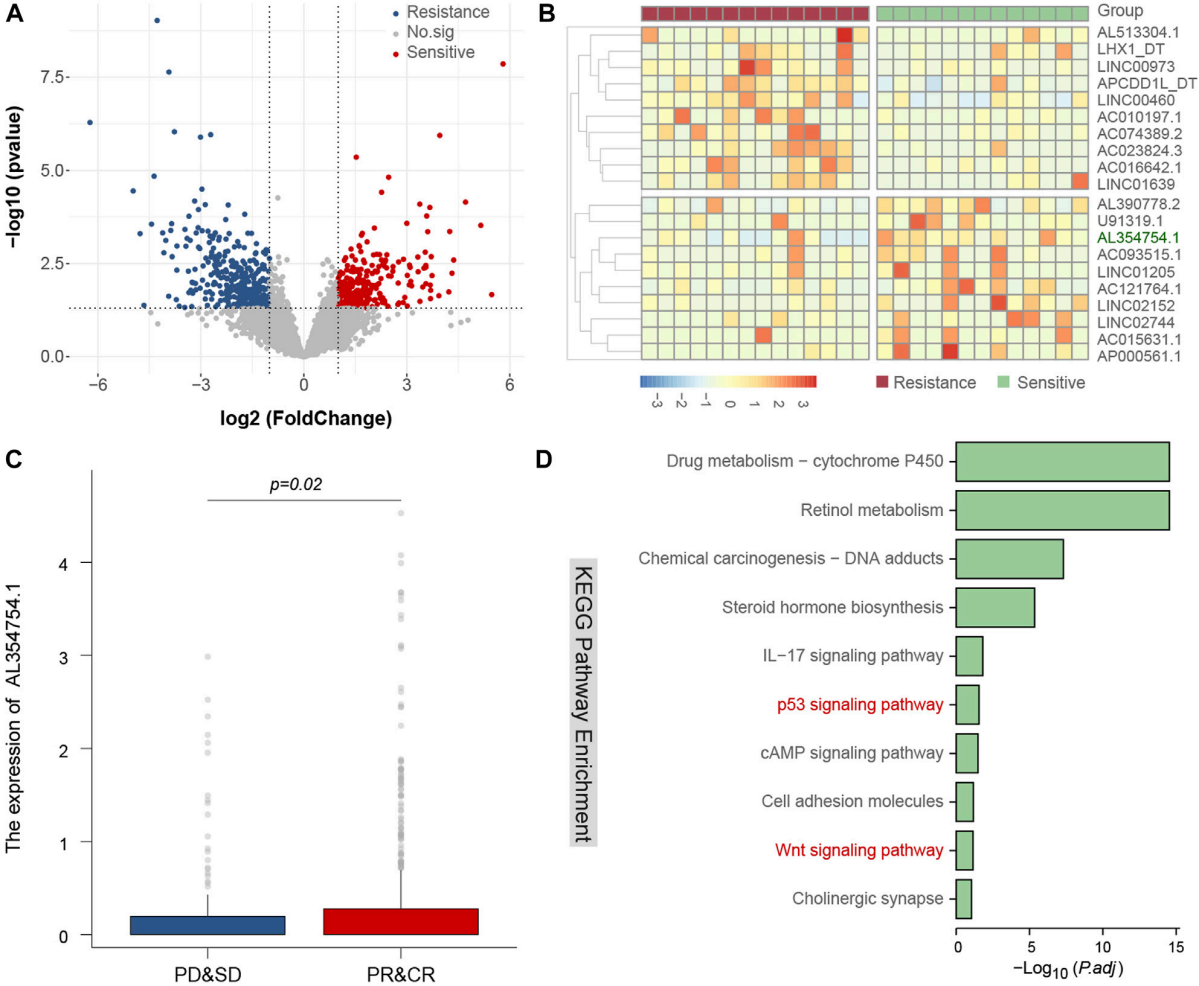
Identify related lncRNAs and candidate mechanism of radiotherapy response. The volcano plot of different expression lncRNAs between radiotherapy resistance and sensitive patients **(A)**, the heatmap of different expression lncRNAs between radiotherapy resistance and sensitive patients, and AL354754.1 is a key lncRNA and could predict radiotherapy response **(B,C)**, and the candidate mechanism of this lncRNA may be involved in classical p53 and Wnt signaling pathway **(D)**.

To explore the candidate mechanism of ISB therapy response, we download public genetic data and conduct different expression Lncrnas analysis and pathway analysis to discover radiotherapy related sensitive or resistance lncRNAs and pathways, we found that AL654754.1 is a key lncRNA with radiotherapy response, and it also include in classical p53 and Wnt signaling pathway (Figure 3).

## Discussion and conclusion

MET amplification, which includes Met exon 14 skipping mutation and amplification, is a crucial driver of non-small cell lung cancer (NSCLC) (Fujino et al., 2020). In patients with inoperable advanced MET aberrations, targeted therapy is the preferred treatment option. Currently, tyrosine kinase inhibitors (TKI) such as capmatinib and crizotinib (Malik et al., 2020) are commonly used for targeted therapy (Malik et al., 2020). However, as seen with other inhibitors, drug resistance is inevitable. Additionally, even though the majority of patients with MET mutations express PD-L1, the tumor mutation burden (TMB) is low, and the overall response rate to immunotherapy is modest (Joshua et al., 2018).

In this case report, we achieved a good therapeutic effect with the combination of ISB and immunotherapy for patients with advanced NSCLC. However, the patient’s chest CT examination revealed a large exudation shadow, which was considered to be radiation pneumonia or immune pneumonia and gradually improved after treatment with methylprednisolone. ISB, as a favorable salvage treatment, may be safe and associated with few complications during the treatment of chest tumors (Ji et al., 2020). Additionally, radiation pneumonia may be associated with autoimmune status or infectious pneumonia (Li et al., 2016); (Cousin et al., 2021). In any case, when combining treatments, we should be aware of any overlap with other infectious or inflammatory diseases, and differential diagnosis and early treatment are especially important. Immune-related pneumonia (IRP) is a side effect associated with immunotherapy, especially treatment with immune checkpoint inhibitors. The prevention of IRP mainly lies in the appropriate use of immunotherapy, including correctly selecting suitable patients and timely and effectively managing potential side effects. During treatment, doctors and patients should maintain close communication, detect and deal with any symptoms as early as possible. The diagnosis of IRP mainly relies on imaging and pathology. Imaging (such as CT scans) can reveal inflammation in the lungs, while pathological examination (such as lung biopsy) can determine the type and extent of inflammation. However, diagnosing IRP also requires excluding other possible causes, such as infections and lung cancer. The treatment of IRP mainly relies on corticosteroids (such as prednisone) because these drugs can suppress the excessive reaction of the immune system. However, if corticosteroids are ineffective, other immunosuppressants may be needed. Radiation-induced pneumonia (RIP) is a type of pneumonia caused by radiotherapy. The prevention of RIP mainly lies in accurate radiotherapy planning and execution, as well as timely supportive treatment. Precise radiotherapy can minimize damage to normal lung tissue, and supportive treatment (such as oxygen therapy) can help patients relieve symptoms. The diagnosis of RIP also relies on imaging, as it can show inflammation and fibrosis in the lungs. The treatment of RIP mainly depends on corticosteroids because these drugs can suppress the excessive reaction of the immune system. However, if corticosteroids are ineffective, other drugs, such as antifibrotic drugs, may be needed.

ISB is a common treatment for advanced or recurrent NSCLC patients (Wu et al., 2018). Compared with traditional radiation therapies, ISB has several advantages, including small size, high local radiation dose, and short half-life decay time (Zhang et al., 2020). ISB can continuously release low radiation doses to kill tumor cells in different proliferation cycles and decay quickly to reduce the probability of complications (Subir et al., 1993; John et al., 2023). Additionally, ISB can effectively relieve cancer pain and improve patient’s quality of life (Fan and Zhou, 2020). Similar to other radiotherapies, ISB is a local treatment that needs to be combined with other systemic therapies to maximize its anti-tumor effect in patients with advanced tumors. Previous studies have shown that combining radiotherapy with immunotherapy can increase antigen release, destroy tumor stroma, and produce an abscopal effect (Gameiro et al., 2014; Valkenburg et al., 2018). Furthermore, local radiotherapy may reverse immune checkpoint inhibitors (ICIs) resistance after immunotherapy failure (Yuan et al., 2017). Low-dose radiotherapy can reverse tumor immune desertification, and resistance to immunotherapy, and reconstruct the tumor immune microenvironment (Herrera et al., 2022).

ISB can damage DNA, preventing cancer cells from growing and dividing. This may affect various signaling pathways, such as the DNA damage response pathway. The potential mechanism is that ISB can directly cause DNA double-strand breaks, triggering a DNA damage response (DDR). DDR involves many different signaling pathways, such as the p53 pathway, the ATM/ATR pathway, and so on. If these pathways are activated, it can lead to cell cycle arrest, DNA repair, or cell death. In our research, we also confirmed that p53 pathway has been changed, suggesting that abnormal activation of the p53 pathway might be a key breakthrough point to increase the ISB sensitivity of patients.

In this case report, we achieved a good therapeutic effect with the combination of ISB and immunotherapy for patients with advanced NSCLC. However, the patient’s chest CT examination revealed a large exudation shadow, which was considered to be radiation pneumonia or immune pneumonia and gradually improved after treatment with methylprednisolone. ISB, as a favorable salvage treatment, may be safe and associated with few complications during the treatment of chest tumors (Ji et al., 2020). Additionally, radiation pneumonia may be associated with autoimmune status or infectious pneumonia (Li et al., 2016). Other studies have shown that ICIs are a potential risk factor for radiation recall pneumonitis and can trigger delayed radiation-induced lung toxicity (Cousin et al., 2021). In any case, when combining treatments, we should be aware of any overlap with other infectious or inflammatory diseases, and differential diagnosis and early treatment are especially important.

In conclusion, we believe that the combination of ISB and immunotherapy can be a promising treatment option for patients with advanced non-small cell lung cancer with MET mutations. However, it is essential to monitor and manage potential complications associated with this treatment approach. Further validation through large randomized clinical trials is needed to confirm its efficacy and safety (Lyczek et al., 2012; Lyczek et al., 2012).

## Data Availability

The original contributions presented in the study are included in the article/Supplementary Material, further inquiries can be directed to the corresponding author.

## Abbreviation

AJCC, American Joint Committee on Cancer; CT, computed tomography; FDA, Food and Drug Administration; IC, immune cells; ICIs, immune checkpoint inhibitors; ISB, I-125 seeds brachytherapy; MET, Cellular-mesenchymal to epithelial transition factor; NSCLC, non-small cell lung cancer; NCCN, National Comprehensive Cancer Network; ORR, objective response rate; PFS, progression-free survival; PD-1, programmed death ligand 1; PD-L1, programmed death ligand 1; PD, progressive disease; PR, partial response; TMB, tumor mutation burden; TKI, tyrosine kinase inhibitors.
